# HDAC6 inhibitor WT161 performs anti-tumor effect on osteosarcoma and synergistically interacts with 5-FU

**DOI:** 10.1042/BSR20203905

**Published:** 2021-04-30

**Authors:** Jun Sun, Wei Wu, Xiaofeng Tang, Feifei Zhang, Cheng Ju, Renfeng Liu, Yiping Liang, Bo Yu, Bin Lv, Yuhong Guo, Duo Zeng, Xuchang Tao, Min Wang, Zhiping Zhang, Changhua Zhang, Xiao-Bin Lv

**Affiliations:** 1 Jiangxi Key Laboratory of Cancer Metastasis and Precision Treatment, Center Laboratory, The Third Affiliated Hospital of Nanchang University, Nanchang 330008, China; 2 College of Pharmacy, Jiangxi University of Traditional Chinese Medicine, Nanchang 330004, China; 3 Hubei Key Laboratory of Economic Forest Germplasm Improvement and Resources Comprehensive Utilization, Huanggang Normal University, Huangzhou, Hubei 438000, China; 4 Nanchang Key Laboratory of Orthopedics, The Third Affiliated Hospital of Nanchang University, Nanchang 330008, China; 5 College of Life Science, Jiangxi Agriculture University, Nanchang 330045, China; 6 Department of Nuclear Medicine, The Third Affiliated Hospital of Nanchang University, Nanchang 330008, China; 7 Department of Orthopedics, Tianjin General Hospital, Tianjin 300052, China

**Keywords:** 5-FU, apoptosis, osteosarcoma, PTEN, synergistic inhibition, WT161

## Abstract

**Background:** WT161, as a selective HDAC6 inhibitor, has been shown to play anti-tumor effects on several kinds of cancers. The aim of the present study is to explore the roles of WT161 in osteosarcoma and its underlying mechanisms.

**Methods:** The anti-proliferative effect of WT161 on osteosarcoma cells was examined using MTT assay and colony formation assay. Cell apoptosis was analyzed using flow cytometer. The synergistic effect was evaluated by isobologram analysis using CompuSyn software. The osteosarcoma xenograft models were established to evaluate the anti-proliferative effect of WT161 *in vivo*.

**Results:** WT161 suppressed the cell growth and induced apoptosis of osteosarcoma cells in a dose- and time-dependent manner. Mechanistically, we found that WT161 treatment obviously increased the protein level of PTEN and decreased the phosphorylation level of protein kinase-B (AKT). More importantly, WT161 showed synergistic inhibition with 5-FU on osteosarcoma cells *in vitro* and *in vivo*.

**Conclusions:** These results indicate that WT161 inhibits the growth of osteosarcoma through PTEN and has a synergistic efficiency with 5-FU.

## Background

Osteosarcoma is the most common primary solid malignant tumor in bones, which is defined as the presence of malignant mesenchymal cells that produce bone-like and/or immature bones [1]. The incidence of osteosarcoma in the general population is 2–3 million per year. However the incidence at puberty is higher, with the highest incidence between 15 and 19 years, reaching 8–11 million per year. In addition, osteosarcoma is usually accompanied with early metastasis, and the vast majority are lung metastases. Ninety percent of osteosarcoma patients die of lung metastasis after multiple chemotherapy [2,3]. Therefore, it is particularly urgent to develop new strategies for the treatment of osteosarcoma.

In recent years, HDAC inhibitors have become a new strategy for the treatment of tumors [4]. More and more HDAC inhibitors have been developed for the treatment of tumors. Recently, WT161, as a selective HDAC6 inhibitor, has been shown to play anti-tumor effects on multiple myeloma, retinoblastoma and breast cancer [5,6]. As a crucial factor regulating acetylation and deacetylation, HDAC6 was reported to be up-regulated in multiple tumors [7–9]. While HDAC6 shows important roles in deacetylating histones, recent reports have identified several critical non-histone protein substrates for HDAC6, including α-tubulin, heat shock protein 90 (HSP90), and PTEN. HDAC6 deacetylates PTEN at K163 and promotes the membrane translocation of PTEN, which activates tumor suppressing function of PTEN.

PTEN is an effective tumor suppressor. Its loss of function is often observed in hereditary and sporadic tumors, and it is also one of the most common mutated tumor suppressors in human cancers. PTEN has both phosphatase activity and non-enzymatic functions (scaffolding) in cells. It relies on these functions to control various biological processes, including genome stability, cell survival, migration, proliferation, and metabolism [10–12]. Even a small decline in the expression level or enzyme activity of PTEN could lead to cancer susceptibility and tumor development. Several studies have found that the absence of PTEN can enhance the growth and lung metastasis of osteosarcoma [13]. In this study, we sought to explore the role of WT161 in osteosarcoma and we found that WT161 suppressed the cell growth of osteosarcoma and synergistically interacted with 5-FU through increasing PTEN expression.

## Methods

### Cell culture and chemicals

The human osteosarcoma cell lines U20S and MG63 were kindly provided by Professor Kang (Sun Yat-sen University Cancer Center, Guangzhou, China) [14]. The U20S and MG63 cells were cultured in DMEM (Thermo Fisher Scientific, Waltham, MA, U.S.A.) medium supplied with 10% fetal bovine serum (FBS) (Thermo Fisher Scientific, Waltham, MA, U.S.A.) at 37°C in 5% CO_2_ atmosphere. WT161 was purchased from MedChemExpress (U.S.A.) and prepared in dimethyl sulfoxide (DMSO) (Sigma–Aldrich, St. Louis, MO, U.S.A.).

### siRNA transfection

PTEN siRNA (GGUGUAAUGAUAUGUGCAUdTdT) was previously validated and synthesized by GenePharma company (Shanghai, China) [15]. An siRNA targeting GFP was used as a negative control and was obtained from GenePharma. Transfection of siRNA was performed using LipoRNAMAX (Thermo Fisher Scientific, Waltham, MA, U.S.A.) according to the manufacturer’s instructions.

### qRT-PCR

Total RNA was extracted using TRIzol (Thermo Fisher, U.S.A.) according to the manufacturer’s instructions. cDNA was obtained using PrimeScript RT Master Mix (Takara, Japan). qRT-PCR was performed using TB Green® Fast qPCR Mix according to the manufacturer’s instructions. The qRT-PCR primer: forward 5′-CGGCAGCATCAAATGTTTCAG-3′, and reverse 5′-AACTGGCAGGTAGAAGGCAACTC-3′ for PTEN; and forward 5′-GAGAGAAACCCGGGAGGCTA-3′, and reverse 5′-TCACCTTCCCCATGGTGTCT-3′ for GAPDH.

### Cell viability assay

The cell viability was examined using CCK-8 kits (Apexbio, Houston, U.S.A.). Briefly, cells were plated into 96-well plates at a density of 5000 cells/well for 24 h and then treated with chemicals for different times. The CCK-8 solution was added to each well at a ratio of 1:5 to medium and cultured for 1 h. Then the OD value was evaluated at 450 nm. The relative OD value = OD value of experimental well/OD value of control well.

### Colony formation

Cells plated into six-well plates at a density of 500 cells/well for 24 h were added with chemicals and cultured for 10 days with the replacement of fresh medium every 2 days. Then the cells were carefully rinsed twice with PBS and immobilized with methanol for 30 min. After the immobilization, the cells were stained with 2 ml of 0.1% Crystal Violet for 30 min and washed three times with PBS. The colonies were photographed and counted under microscope.

### Apoptosis assay

The apoptosis assay was performed using Annexin V-FITC/PI apoptosis detection kits (BD Biosciences, U.S.A.) according to the manufacturer’s instructions. Briefly, cells were trypsinized and suspended in suspension buffer. Then the Annexin V-FITC and PI were added separately into the cell suspension and incubated for 30 min at room temperature. The cell apoptosis was detected and analyzed by flow cytometry.

### Western blotting

Cells were lysed with RIPA (50 mM Tris/HCl, 150 mM NaCl, 5 mM EDTA, 0.5% Nonidet P-40) buffer containing a protease and phosphatase inhibitor cocktail (Calbiochem, San Diego, CA) for 30 min and were centrifuged at 14000 rpm at 4°C for 25 min. The supernatants were added with one-fourth volume of 5× SDS loading buffer and boiled for 10 min. Ten micrograms of protein from each sample was separated by SDS/PAGE. Then, the proteins were transferred to a PVDF membrane. After blocking with 5% fat-free milk for 1 h, the membrane was sequentially probed with primary antibody and secondary antibody. Antibody detection was performed with a chemiluminescence kit (Beyotime LTD, Shanghai, China).

### Xenograft murine model

Twenty-four female nude mice aged 4–6 weeks were prepared for tumor implantation. Each nude mouse was inoculated subcutaneously with 3 × 10^6^ osteosarcoma cells in 200 μl PBS buffer. The xenograft tumor formation was monitored everyday. One week after implantation, when the tumors became palpable at a diameter of approximately 2 mm, the nude mice were randomly divided into four groups with six mice in each group and PBS, WT161 (80 mg/kg), 5-FU (5 mg/kg), or WT161 and 5-FU combination were injected intraperitoneally once a day, respectively. The volumes of the tumors were measured once daily. At day 14, mice were subjected to CO_2_ asphyxiation and apnea was confirmed. They were then subjected to cervical dislocation for secondary assurances. Then the primary tumors were carefully removed, imaged, and weighed. The tumor volumes were calculated using the formula as tumor volume = 0.5 × tumor length × (tumor width)^2^. The animal experiments were carried out in Nanchang University, were approved by the Medical Ethics Committee of The Third Affiliate Hospital of Nanchang University, and were carried out in accordance with the Principles of Laboratory Animal Care (NIH publication no. 85Y23, revised 1996).

### Evaluation of the synergistic effect

Evaluation of synergistic effect between two chemicals was conducted as described previously [16]. Briefly, using drug concentrations based on the half maximal inhibitory concentration (IC_50_) value of each drug as a single drug to produce growth inhibition of approx. 10–90%. When the drugs are used in combination, the drug concentration of the two drugs should be kept at a certain ratio. The combination index (CI) was calculated by the CompuSyn software (Biosoft).

### Statistical analysis

We used statistical software SPSS 16 to analyze the experimental data. Results are shown as the mean ± standard deviation (SD). LSD test was used to detect the statistical difference between experimental groups. A *P*-value less than 0.05 was considered statistically significant.

## Results

### WT161 suppresses the cell growth of osteosarcoma cells

**Figure 1:**
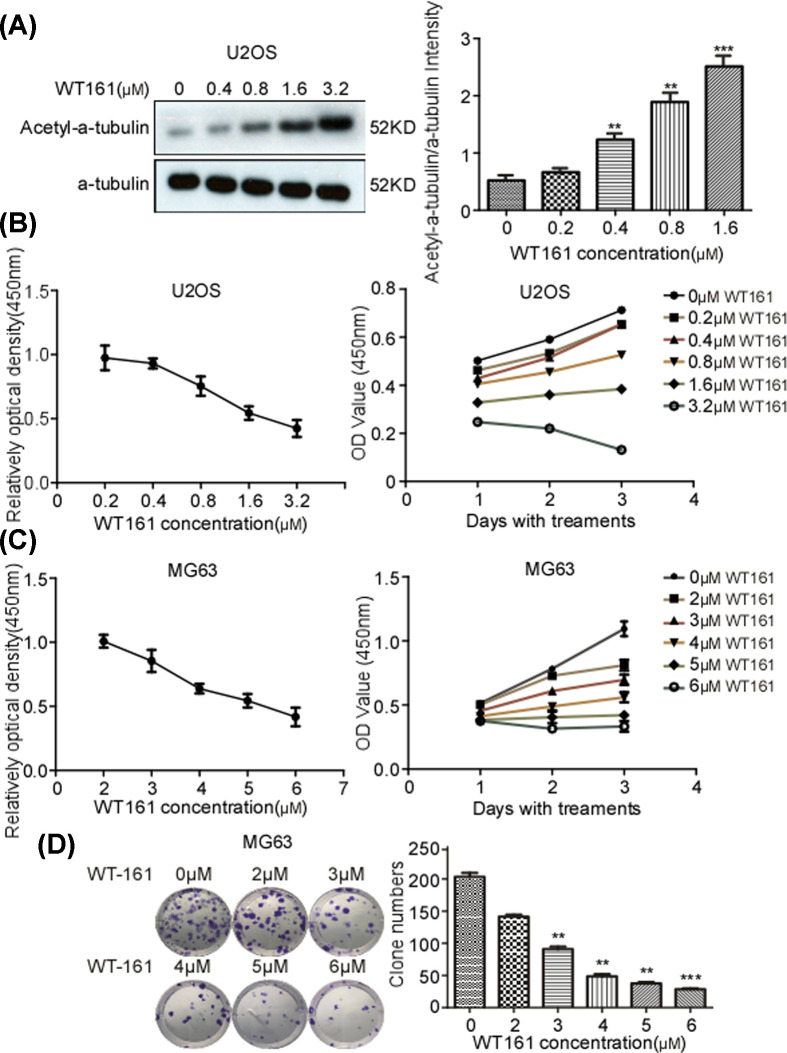
WT161 suppresses the cell growth of osteosarcoma cells (**A**) U2OS cells were subjected to various concentrations of (0–1.6 µM) WT161 for 48 h and the acetyl-α-tubulin was examined. (**B**) U2OS cells were treated with various concentrations of WT161 for 48 h (left) or different days (right) and the cell growth was assessed using CCK-8 assay. (**C**) MG63 cells were treated with various concentrations of WT161 for 48 h (left) or different days (right) and the cell growth was accessed using CCK-8 assay. (**D**) MG63 cells were inoculated into six-well plates (1000 cells/well). Each well was treated with different concentrations of WT161 (0–6 µM) for two weeks. The cells were stained with 0.1% Crystal Violet dye overnight. *n*=3, **P<0.01, ***P<0.001.

To investigate the roles of WT161 in osteosarcoma cells, we treated U2OS and MG63 cells with a series concentration of WT161. The enhancement of acetyl-α-tubulin level upon WT161 treatment indicated the efficiency of WT161 (Figure 1A). A viability assay showed that WT161 suppressed the cell growth of U2OS and MG63 cells in a dose- and time-dependent manner (Figure 1B,C). In addition, a colony formation assay showed that WT161 inhibits the colony formation of MG63 cells in a dose-dependent manner (Figure 1D).

### WT161 induces the apoptosis of osteosarcoma cells

**Figure 2:**
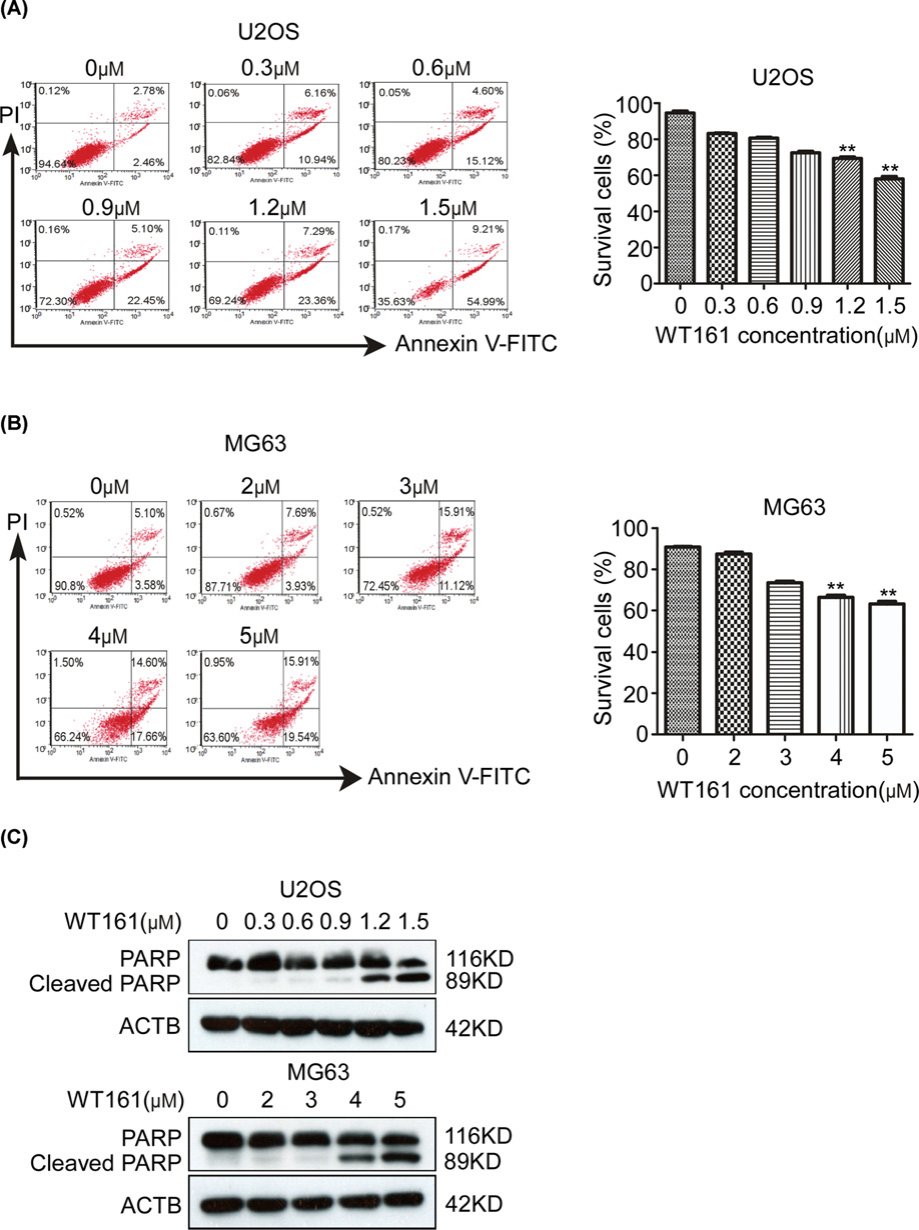
WT161 treatment induces the apoptosis of osteosarcoma cells (**A**) U2OS cells were subjected to various concentrations of WT161 for 48 h. The cell apoptosis was assessed by flow cytometry. *n*=3, **P<0.01. (**B**) MG63 cells were subjected to various concentrations of WT161 for 48 h. The cell apoptosis was assessed by flow cytometry. *n*=3, **P<0.01. (**C**) The protein expression levels of PARP and cleaved PARP were tested in U2OS and MG63 cells treated with WT161 for 48 h using the Western blot analysis. *n*=3, **P<0.01.

In order to determine whether WT161 affects the apoptosis of osteosarcoma cells, we examined the apoptosis rate of osteosarcoma cells upon WT161 treatment through flow cytometry. As shown in Figure 2A,B, the apoptotic cells were elevated in U2OS and MG63 cells upon WT161 treatment in a dose-dependent manner. In addition, the cleaved PARP, a marker indicating the cell apoptosis, was increased upon WT161 treatment (Figure 2C). These results indicate that WT161 improves the apoptosis of osteosarcoma.

### WT161 increases the apoptosis of osteosarcoma cells mainly through regulating PTEN/protein kinase-B signaling pathway

**Figure 3:**
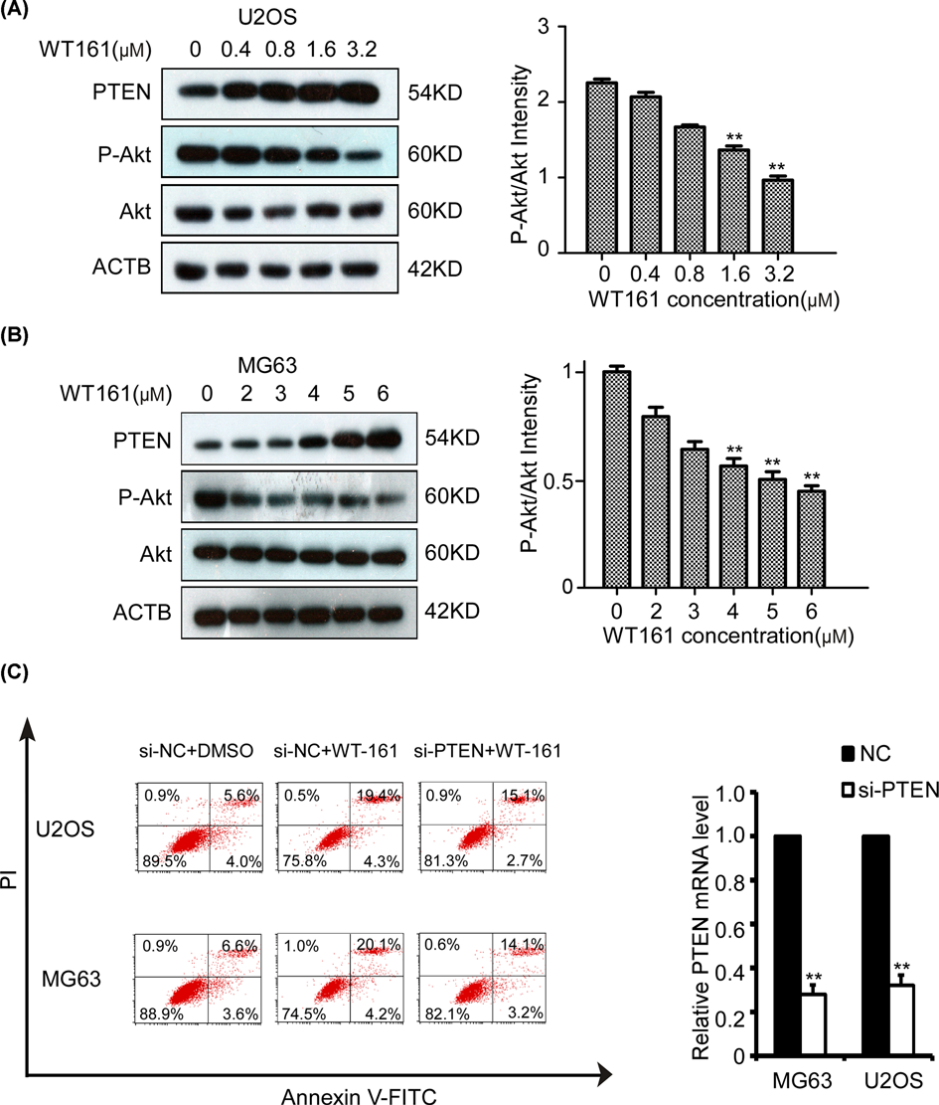
WT161 increases the apoptosis of osteosarcoma cells mainly through regulating PTEN/AKT signaling pathway (**A**) U2OS cells were treated with WT161 for 48 h and the protein expression levels of PTEN, P-Akt and Akt were tested using Western blot. *n*=3, **P<0.01. (**B**) MG63 cells were treated with WT161 for 48 h and the protein expression levels of PTEN, P-Akt and Akt were tested using Western blot. *n*=3, **P<0.01. (**C**) Silencing of PTEN reduces the apoptosis of osteosarcoma cells. U2OS and MG63 cells transfected with PTEN siRNA or negative control for 24 h were treated with WT161 for 48 h and the apoptotic cells were evaluated by flow cytometry.

Previous studies have demonstrated that PTEN/protein kinase-B (AKT) signaling pathway was usually overactivated in osteosarcoma and promoted the tumorigenesis and progression of osteosarcoma [17,18]. In addition, HDAC6 was reported to be implicated in the translocation and activation of PTEN. Therefore, we sought to determine whether PTEN/AKT signaling pathway mediated the killing effects of WT161 on osteosarcoma cells. Firstly, WT161 treatment obviously increased the protein expression level of PTEN and decreased the phosphorylation level of AKT in both U2OS and MG63 cells (Figure 3A,B). Importantly, silence of PTEN attenuated WT161-induced cell apoptosis of osteosarcoma cells (Figure 3C). These results indicate that WT161 increases the apoptosis of osteosarcoma cells mainly through the regulation of PTEN/AKT signaling pathway.

### WT161 shows synergistic effects on osteosarcoma cells combined with 5-FU

**Figure 4:**
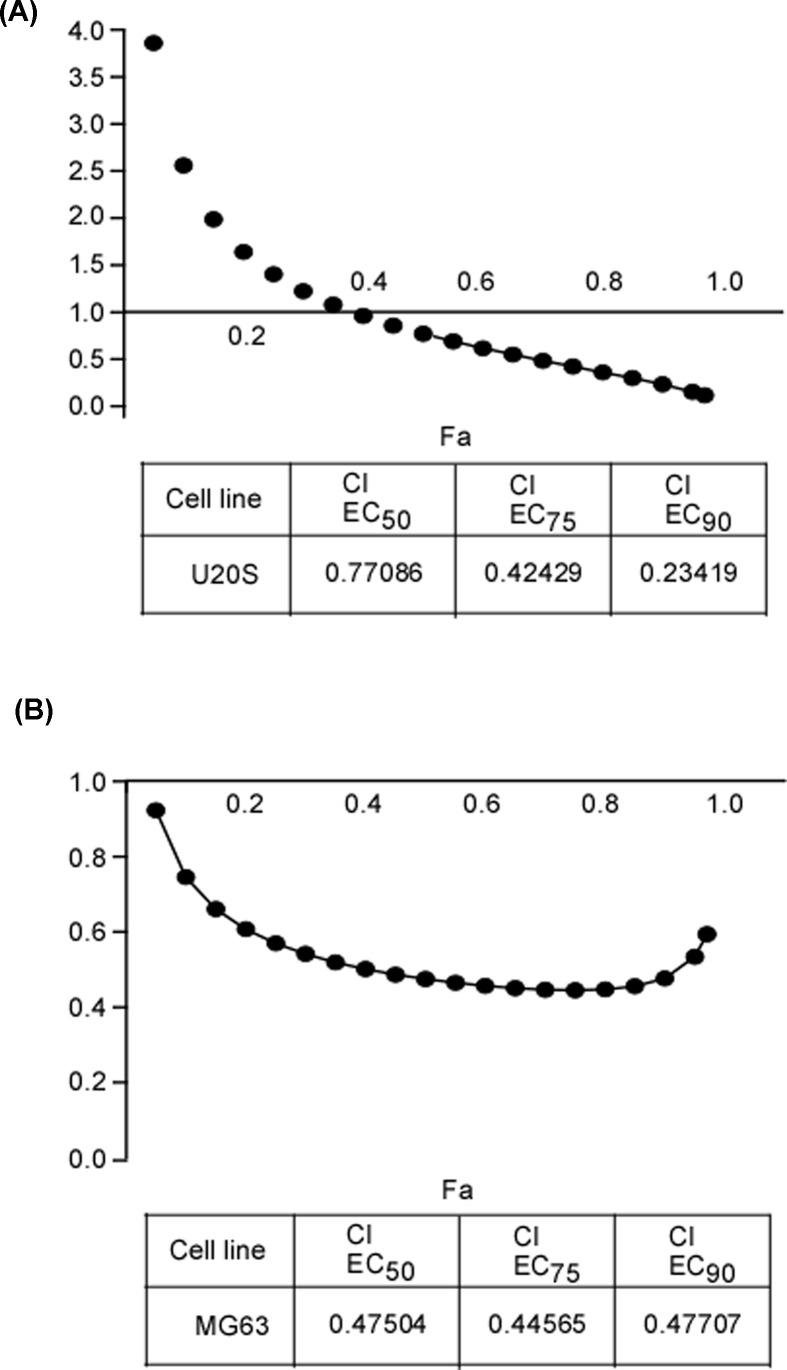
WT161 shows synergistic inhibitory effects on osteosarcoma cells combined with 5-FU (**A**) U2OS cells were subjected to 5-FU alone or in combined with WT161. The synergistic effect of various concentration ranges of WT161 and 5-FU were shown using CDI. (**B**) MG63 cells were subjected to 5-FU alone or in combination with WT161. The synergistic effect of various concentration ranges of WT161 and 5-FU were shown using CDI.

Several studies have found that PTEN/phosphatidylinositol 3-kinase (PI3K)/AKT signaling pathway was related to the sensitivity of cancer cells to chemotherapy especially 5-FU [19–23]. Thus, we explored whether WT161 synergistically inhibits the cell growth of osteosarcoma cells combined with 5-FU. Indeed, the results showed the CI values for WT161 and 5-FU were lower than 0.7, indicating that a synergistic effects of WT161 and 5-FU on osteosarcoma cells (Figure 4A,B).

### WT161 shows synergistic effects on the suppression of osteosarcoma combined with 5-FU in a mouse xenograft model

**Figure 5:**
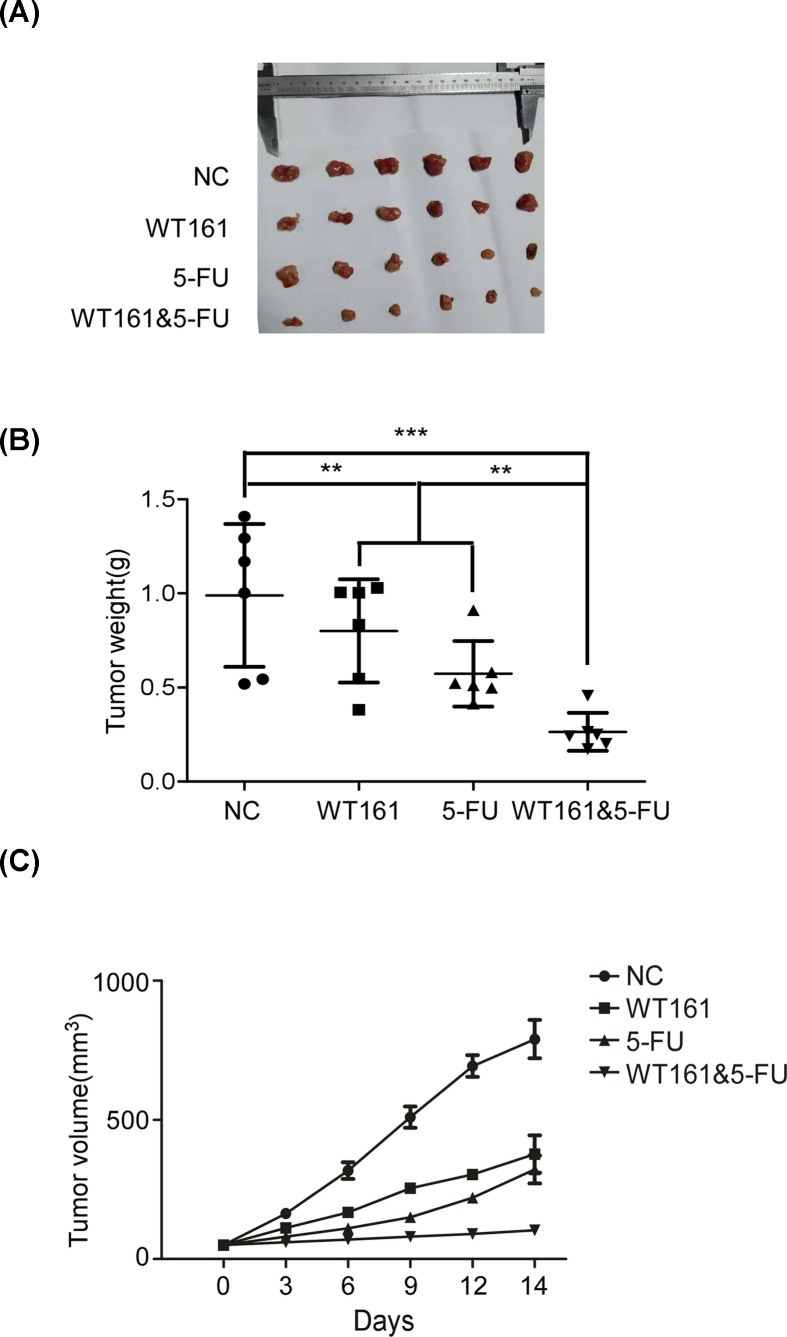
WT161 shows synergistic inhibitory effects on osteosarcoma cells combined with 5-FU in a mouse xenograft model (**A**) Xenograph tumors dissected from node mice. (**B**) Statistical analysis of the weight of each experimental group. (**C**) The growth of xenograph tumors of each experimental group. **P<0.01, ***P<0.001.

To further confirm the effect of WT161 on osteosarcoma, we evaluated the anti-proliferative effect and synergistic effects of WT161 and 5-FU in xenograft murine model. As shown in Figure 5A,B, WT161 and 5-FU significantly inhibited the growth and weight of osteosarcoma tumor. In addition, compared with WT161-administration and 5-FU-administration group, WT161 and 5-FU-administration group showed much more suppressing effect on the growth of osteosarcoma xenograft (Figure 5C). This result further illustrates that WT161 and the clinical chemotherapeutic drug 5-FU have a synergistic efficiency in suppression of osteosarcoma.

## Discussion

Osteosarcoma is the most common primary malignant bone tumor and is usually accompanied with early metastasis, especially lung metastases. Ninety percent of osteosarcoma patients died of lung metastasis after a variety of chemotherapies [24]. At present, chemotherapy is still the most important method for the treatment of osteosarcoma, but the rapid occurrence of resistance and serious adverse effects limit the application of chemotherapy. Therefore, it is very important to develop methods with good curative effect and little side effect [2,3].

PI3K/AKT signaling pathway is one of the most important intracellular pathways, which regulates cell survival, growth, differentiation, metabolism, and cytoskeletal reorganization. The main proteins involved in this signaling pathway are PI3K and Akt [25–27]. At present, growing studies have shown that PI3K/AKT signaling pathway is often over-activated in osteosarcoma and promotes the occurrence and development of tumors, including cell proliferation, invasion, cell cycle progression, apoptosis inhibition, angiogenesis, metastasis, and chemical resistance. Inhibiting this pathway through small molecule compounds have also become an attractive treatment for osteosarcoma [18]. PTEN acts as a major negative regulator of the PI3K/Akt signaling pathway, and loss of PTEN activity is often found in osteosarcoma [17]. Several groups reported that treatment of osteosarcoma with 5-azacytidine significantly increased the expression of PTEN and down-regulated AKT signaling, causing apoptosis of osteosarcoma cells [28]. In this study, we found that WT161 increased PTEN expression and inhibited the downstream PI3K/Akt signal pathway, thus inducing apoptosis of osteosarcoma cells.

Recently, although it has made progression in the adjuvant chemotherapy for osteosarcoma chemotherapy and the survival rate has been significantly improved, the overall prognosis is still poor [18]. Therefore, it is still urgent to develop novel treatment approach for the treatment of osteosarcoma patients. It was reported that up-regulation of PTEN or inactivation of its downstream signal PI3K/AKT pathway increased the killing effect of 5-FU on cancer cells and overcame drug resistance [19–23]. Based on our results that WT161 induced the up-regulation of PTEN, we speculated that WT161 and 5-FU may have a synergistic effect on osteosarcoma. As expected, WT161 obviously enhance the cytotoxicity of the 5-FU on osteosarcoma *in vitro* and *in vivo*. These results indicate that the combined application of WT161 and 5-FU may be an effective strategy for the treatment of osteosarcoma in the future.

## Conclusion

In conclusion, we demonstrated that WT161 inhibits the growth of osteosarcoma cells in a dose- and time-dependent manner and increases its apoptosis mainly by increasing the protein expression of pro-apoptotic protein PTEN and subsequently PI3K/Akt inactivation. In addition, it showed significant synergy with 5-FU in killing osteosarcoma cells *in vitro* and *in vivo* assay. Our findings provide evidence that WT161 might be a promising agent against osteosarcoma.

## Data Availability

All data are included in the paper.

## Abbreviations

AKT/Akt: protein kinase-B
CI: combination index
HDAC: histone deacetylase
MTT: 3-(4,5-Dimethylthiazol-2-yl)-2,5-diphenyltetrazolium bromide
PI: propidium iodide
PI3K: phosphatidylinositol 3-kinase
